# Prevention of post-operative complications by using a HMG-CoA reductase inhibitor in patients undergoing one-lung ventilation for non-cardiac surgery: study protocol for a randomised controlled trial

**DOI:** 10.1186/s13063-018-3078-5

**Published:** 2018-12-18

**Authors:** Murali Shyamsundar, Cecilia O’Kane, Gavin D. Perkins, Gavin Kennedy, Christina Campbell, Ashley Agus, Glenn Phair, Danny McAuley

**Affiliations:** 1 Centre for Experimental Medicine, Queen’s University Belfast, Belfast, BT9 7BL UK; 2 Warwick Trials Unit, University of Warwick, Coventry, CV4 7AL UK; 3 Northern Ireland Clinical Trials Unit, 1st Floor Elliott Dynes Building, Royal Hospitals, Belfast, BT12 6BA UK

**Keywords:** Simvastatin, Post-operative pulmonary complication, Post-operative myocardial infarction, Acute respiratory distress syndrome, One-lung ventilation, Oesophagectomy, Lobectomy, Pneumonectomy

## Abstract

**Background:**

Postoperative pulmonary complications (PPC) and peri-operative myocardial infarction (MI) have a significant impact on the long-term mortality of surgical patients. Patients undergoing one-lung ventilation (OLV) for surgery are at a high risk of developing these complications. These complications could be associated with intensive care unit (ICU) admissions and longer hospital stay with associated resource and economic burden. Simvastatin, a HMG-CoA reductase enzyme inhibitor has been shown to have pleiotropic anti-inflammatory effects as well as being endothelial protective. The benefits of statins have been shown in various observational studies and in small proof-of-concept studies. There is an urgent need for a well-designed, large clinical trial powered to detect clinical outcomes. The Prevention HARP 2 trial will test the hypothesis ‘simvastatin 80 mg when compared to placebo will reduce cardiac and pulmonary complications in patients undergoing elective oesophagectomy, lobectomy or pneumonectomy’.

**Methods/design:**

The Prevention HARP 2 trial is a UK multi-centre, randomised, double-blind, placebo-controlled trial. Adult patients undergoing elective oesophagectomy, lobectomy or pneumonectomy will be eligible. Patients who are already on statins will be excluded from this trial. Patients will be randomised to receive simvastatin 80 mg or matched placebo for 4 days pre surgery and for up to 7 days post surgery. The primary outcome is a composite outcome of PPC and MI within 7 days post surgery. Various secondary outcome measures including clinical outcomes, safety outcomes and health economic outcomes will be collected. The study aims to recruit 452 patients in total across 12 UK sites.

**Discussion:**

The results of the Prevention HARP 2 trial should add to our understanding of the benefits of peri-operative statins and influence clinical decision-making. Analysis of blood and urine samples from the patients will provide insight into the mechanism of simvastatin action.

**Trial registration:**

International Standard Randomised Controlled Trials registry, ID: ISRCTN48095567. Registered on 11 November 2016.

**Electronic supplementary material:**

The online version of this article (10.1186/s13063-018-3078-5) contains supplementary material, which is available to authorized users.

## Background

One-lung ventilation (OLV) is an anaesthetic technique used commonly in surgeries like oesophagectomy, lobectomy and pneumonectomy. These surgeries are associated with high rates of post-operative pulmonary complications (PPC) with rates between 13 and 43% in the literature [1–3]. The myocardial infarction (MI), including myocardial infarction after non-cardiac surgery (MINS), rate is approximately 8% with a higher rate of up to 16% in patients undergoing thoracotomy for non-cardiac surgery [4, 5]. Post-operative pulmonary complications and MI are common and devastating clinical conditions with high morbidity and mortality, intensive care unit (ICU) admissions and length of stay [6–8]. Cardiac and respiratory complications have both immediate and long-standing resource implications that include an increase in ventilator usage, critical care support and on-going rehabilitation needs in the community post discharge [3, 7, 9]. The occurrence of post-operative complications within 30 days is a strong determinant of both short-term and long-term mortality [10].

This highlights the need for multi-centre, randomised, placebo-controlled trials of therapies aimed at reducing the incidence of PPC, acute respiratory distress syndrome (ARDS) and MI to improve patient outcomes.

### Statins can modulate mechanisms important in the pathogenesis of pulmonary and cardiac complications

Statins have significant immunomodulatory properties in addition to reducing plasma cholesterol levels. Pulmonary complications, including ARDS, are inflammatory conditions driven by inflammatory cells such as neutrophils [11] and macrophages [12]. Hydroxy-methylglutaryl coenzyme A reductase (HMG-CoA) inhibition with statins is a promising new therapeutic option since statins modulate a number of the underlying processes described in the development of pulmonary and cardiac complications. Statins have diverse anti-inflammatory properties [12] that reduce pulmonary inflammation and improve atherosclerotic plaque stability [13]. In an inhaled lipopolysaccharide (LPS) model of pulmonary inflammation and injury, simvastatin 80 mg significantly reduced pulmonary neutrophil infiltrate and both cytokines and tissue-degrading proteases in bronchoalveolar lavage, compared with placebo [12].

One-lung ventilation leading to pulmonary inflammation is one of the putative mechanisms driving PPC rates and atherosclerotic plaque instability is a key mechanism for post-operative MI. Simvastatin has been shown to be beneficial in reducing pulmonary inflammation and injury in human in-vivo models of pulmonary injury [12] as well as in a single-centre trial in patients undergoing oesophagectomy [14] while conferring protection against MI by increasing plaque stability, improving endothelial function [15]. There is a strong biological rationale for using simvastatin to reduce the incidence of post-operative inflammatory complications, such as PPC, ARDS and MI, that form the composite primary outcome for this trial.

### Studies support a multi-centre clinical trial of statin to prevent pulmonary and cardiac complications

A recent meta-analysis, which included more than 2000 patients undergoing surgery for cardiac, non-cardiac and vascular indications, concluded that pre-operative statin use was associated with a significant reduction in post-operative MI, atrial fibrillation (AF) and length of hospital stay [16]. Peri-operative statin use has been shown to reduce mortality, cardiovascular and respiratory complications in a recent retrospective database analysis involving more than 48,000 propensity-matched pairs of patients undergoing non-cardiac surgery [17]. Simvastatin was the most frequently used statin in most of the studies. In cardiovascular surgery patients, the addition of a statin pre-operatively significantly reduced mortality, AF and MI [18] while in a single-centre prospective observational study of patients undergoing general non-cardiac surgery, non-cardiac complications, including PPC, were reduced [19]. Statins also reduced PPC in patients undergoing pulmonary resection who were randomised to receive atorvastatin 40 mg or placebo prior to surgery [20] but this was a single-centre, underpowered study. Similarly, simvastatin 80 mg administered pre-operatively for 4 days and post-operatively for 7 days significantly reduced pulmonary inflammation in patients undergoing OLV for oesophagectomy [14].

While the above studies demonstrate potential benefit for the use of statins as an anti-inflammatory therapy, in a recently concluded multi-centre trial in patients with ARDS, simvastatin 80 mg did not show any benefit in mortality or ventilatory-free days of organ dysfunction [21] but this is likely secondary to patient heterogeneity where a post-hoc analysis suggests benefit in patients with a hyper-inflammatory phenotype [22]. Similarly, a large multi-centre study investigating the benefit of peri-operative atorvastatin and acute kidney injury following cardiac surgery was stopped early due to futility [23]. The LOAD trial, which is a peri-operative trial of atorvastatin administered prior to surgery and continued for 7 days post surgery in non-cardiac surgery, did not show any reduction in the composite outcome of mortality, MI and MINS. There was a 40% non-significant reduction in the primary outcomes in a landmark sensitivity analysis. The lack of effect is possibly due to the short duration of pre-operative statin exposure where an increase by even a day of statin therapy is associated with relative reduction in post-operative MI [24].

These studies demonstrate the conflicting evidence but potential benefits of statins in preventing pulmonary and cardiac complications post surgery, the biological rationale for these effects and the urgent need for an adequately powered multi-centre trial.

### Safety of peri-operative statin use

Statins have been proven to be a well-tolerated class of drugs. Simvastatin 80 mg is within the licensed therapeutic range for the treatment of hypercholesterolaemia. In a study where 2265 patients following an acute coronary syndrome were randomised to receive simvastatin 80 mg, myopathy (creatine kinase) (creatine kinase (CK) > 10 times the upper limit of normal (ULN) associated with muscle symptoms) occurred in only 0.4% and rhabdomyolysis (CK > 10,000 units/L with or without muscle symptoms) in 0.13% [25]. Importantly in this study, follow-up was only at months 1, 4 and 8 and every 4 months thereafter for up to 24 months until trial completion. The data from our proof-of-concept study reassuringly found that simvastatin 80 mg was well tolerated and not associated with increased adverse events (AEs) compared to placebo. There was no difference in CK levels or numbers of patients with a CK > 10 times the ULN between the groups. There were no differences in serum creatinine (SrCr) levels, liver transaminases (alanine transaminase (ALT) and aspartate aminotransferase (AST)) between the groups. No drug-related serious adverse events (SAEs) occurred during the study. Furthermore, the incidence of clinical adverse outcomes, such as ARDS, infections and arrhythmias, was less in the simvastatin-treated group when compared to the placebo group but this study was not powered to detect clinical outcomes [14].

### Hypothesis

This trial will test the hypothesis that simvastatin 80 mg reduces cardiac and pulmonary complications, when compared to placebo, in patients undergoing elective oesophagectomy, lobectomy or pneumonectomy.

## Methods/design

Ethical approval for this trial was provided by South Central – Berkshire Research Ethics Committee. The International Standard Randomised Controlled Trial Registry number for trial registration is ISRCTN48095567. The sponsor organisation for the trial is the Belfast Health and Social Care Trust (BHSCT). The trial is coordinated by the Northern Ireland Clinical Trials Unit (NICTU; www.nictu.hscni.net). The trial is funded by a National Institute for Health Research (NIHR) Clinician Scientist Fellowship administered through the Research and Development Office of Northern Ireland Public Health Agency (Funder reference no. CDV/5137/15). The trial will be carried out in accordance with the Medical Research Council Good Clinical Practice Guidelines, applicable UK legislation and the standard operating procedures (SOPs) of the NICTU. This manuscript was written in concordance with the Standard Protocol Items: Recommendations for Interventional Trials (SPIRIT) guidelines [26]. The trial will be reported in line with the Consolidated Standards of Reporting Trials (CONSORT) 2010 guidelines [27].

### Outcome measures

#### Primary outcome

The primary outcome measure is a composite endpoint of the incidence of ARDS, defined according to the Berlin definition [28], PPC as defined by the Melbourne Group Scale (MGS) [29] and MI as defined by ischaemic chest pain, electrocardiograph changes and a raise in plasma troponin and also by myocardial ischaemia post non-cardiac surgery (MINS) criteria [4] during the first 7 days post-operatively. These endpoints were chosen based on their effect on short-term and long-term outcomes and a biological rationale for simvastatin in modulating these endpoints.

#### Secondary outcomes

Secondary outcomes include clinical outcomes, safety and health economic outcomes (health-related quality of life (HRQoL) and costs).

##### Clinical outcomes

Mortality at day 28 and day 90, ventilator-free days [30], defined as the number of days in the first 28 days following surgery that a patient is free from ventilator assistance, for greater than 48 h; ARDS, PPC and MI within 28 days of surgery or hospital discharge if earlier; AF within 28 days of surgery or hospital discharge if earlier, venous thromboembolism [31] within 28 days of surgery or hospital discharge if earlier and the incidence and nature of any surgical complications.

##### Safety

Creatine kinase > 10 times the ULN (day 0, day 3 and day 7 post surgery) of the local laboratory range; ALT/AST > 5 times the ULN (day 0, day 3 and day 7 post surgery) of the local laboratory range; acute kidney injury defined according to Kidney Disease Improving Global Outcomes guidelines (using change from baseline SrCr) within 7 days of surgery; SAEs, AEs and occurrence of suspected unexpected serious adverse reactions (SUSARs).

##### Health economic outcomes

HRQoL as measured using the EuroQoL-5 Dimension Questionnaire (5-level version) (EQ-5D-5 L) [32] at baseline and at 90 days post surgery; health and social care resource use: length of ICU stay (level-3 care), length of high-dependency unit stay (level-2 care), length of hospital stay, health service contacts up to 90 days post surgery.

##### Exploratory outcomes

Plasma and urine samples will be collected to understand the mechanism of simvastatin action by measuring markers of inflammation and cellular injury. Biomarkers of systemic inflammation (TNFα, IL-8, IL-6, IL-1β) and epithelial and endothelial injury (von-Willebrand factor, surfactant protein-D, receptor for advanced glycation end product) and a marker of systemic endothelial dysfunction (urine albumin to creatinine ratio) and neutrophil activation (myeloperoxidase, neutrophil elastase) will be measured.

#### Eligibility criteria

Patients’ eligibility to take part in the trial will be confirmed by medically qualified member of the research team when they fulfil the following inclusion and exclusion criteria.

Inclusion criteria:

1. Adult patients ≥ 18 years of age undergoing OLV for elective oesophagectomy, lobectomy or pneumonectomy
2. Female subjects must be surgically sterile, or be postmenopausal, or must agree to use effective contraception during the period of the trial and for at least 30 days after completion of treatment. A pregnancy test, measured by urine human chorionic gonadotropin, in women of child-bearing potential will be performed at the pre-operative assessment clinic

Exclusion criteria:

1. Age < 18 years
2. Creatinine kinase ULN (CK) > 5 times ULN range of the local laboratory range
3. Known active liver disease (Child-Pugh score > 11), or abnormal liver function tests: transaminases (AST or ALT) ULN > 3 times ULN range (ULN) of the local laboratory range
4. Renal impairment (calculated SrCr clearance less than 30 mL/min)
5. Inability to take medication enterally pre-operatively
6. Subject-reported lactose intolerance
7. Participation in other intervention trials within 30 days
8. Current treatment with statins
9. Known hypersensitivity to the study medication
10. Previous adverse reaction (AR) to statins
11. Concomitant use of fibrates or other lipid-lowering therapy
12. Concomitant use of itraconazole, ketoconazole, posaconazole, voriconazole, erythromycin, clarithromycin, telithromycin, HIV-protease inhibitors, boceprevir, telaprevir, nefazodone, cobicistat, cyclosporine, danazol, amiodarone, amlodipine, verapamil or diltiazem, fusidic acid and niacin
13. Patients must be able to understand and give signed and dated informed consent indicating that they understand all the pertinent aspects of the trial prior to enrolment
14. Currently pregnant or lactating

### Sample size calculation

The incidence of PPC varies, ranging from 13 to 43% in the literature [2, 3, 33]. The significant variation is at least partly explained by varying criteria used to define PPC. The incidence of ARDS in our proof-of-concept study was 25% in the placebo group [14]. The incidence of MI ranges from 5 to 8% based on the MINS criteria [11, 30]. In patients undergoing thoracic surgery, a recent study has demonstrated a MINS incidence of 16% [8]. For the sample size calculation, the control group event rate is expected to be approximately 25% with a conservative estimate of 15% PPC (including ARDS) and 10% MI by day 7 post surgery.

There are no prospective randomised controlled trials in surgeries utilising the OLV technique in patients to predict the size of the treatment effect on preventing both cardiac and respiratory complications post surgery. In a single-centre study of atorvastatin in patients undergoing elective pulmonary resection, there was a 50% absolute reduction in PPC rate in the atorvastatin-treated group [20]. In patients undergoing vascular surgery, fluvastatin pre treatment reduced the risk of MI from 20 to 10% [13]. In a randomised, placebo-controlled trial, simvastatin pre treatment was associated with a 37% absolute reduction in troponin release [15]. To inform the likely loss to follow-up, previous randomised controlled trials in oesophagectomy patients have experienced a 1–2% loss-to-follow-up rate over 7 days [34, 35].

The preliminary sample size of 203 is based on a two-group chi-square test with 50% relative reduction in incidence of the composite endpoint from a predicted rate of 25% in the control group with 90% power of detecting this difference at *p* = 0.05. The inclusion of a 10% dropout rate gives an overall sample size of 226 per arm or 452 in total. Our proof-of-concept study indicates that 70% of patients would fulfil the criteria for enrolment. In a recent trial of statins in patients with ARDS, the exclusion rate due to prior statin use was 30% with a further 10% refusing assent/consent [36].

**Fig. 1:**
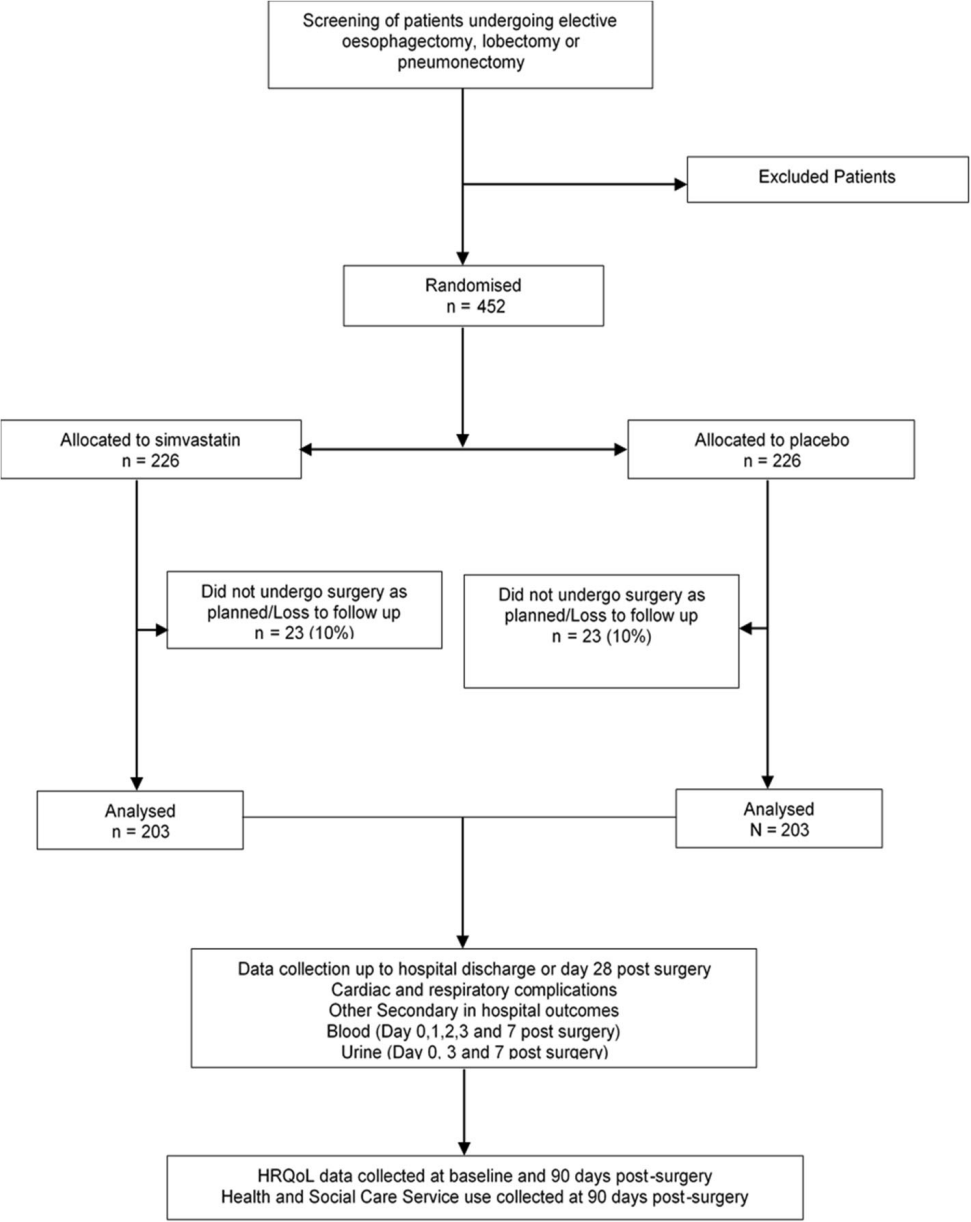
Consolidated Standards of Reporting Trials (CONSORT) diagram

The planned recruitment from 12 UK sites with a conservative recruitment of 50% of eligible patients, which includes a 10% failure to progress to surgery, will enable completion of recruitment in 30 months at the rate of 15 patients/month (Fig. 1: CONSORT diagram).

### Trial conduct

#### Approach to patients and obtaining informed consent

The study will be conducted in hospitals in the UK. An up-to-date list of study sites can be obtained from the NICTU. Patients will be identified through upper gastrointestinal/thoracic oncology multidisciplinary meetings and waiting list offices. Eligible patients will be invited to participate by the research team. If agreeable, written informed consent will be obtained, following a face-to-face discussion about the study. All the members of the research team will undergo Good Clinical Practice (GCP) training, training on study-related documents and on SOPs.

**Fig. 2:**
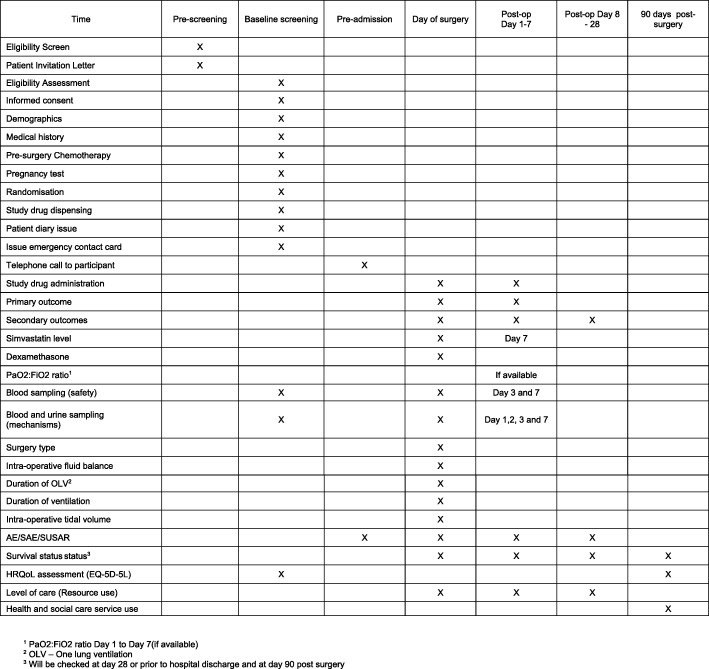
Standard Protocol Items: Recommendations for Interventional Trials (SPIRIT) Checklist

No financial incentive has been planned for trial investigators or participants. No competing interest disclosed by the principal investigators (PIs). The study interventions are detailed in Fig. 2 and in Additional file 1.

#### Sequence generation

Eligible participants will be allocated to intervention or placebo using an automated randomisation system (Sealed Envelopes). After obtaining informed consent, patients will be randomised on a 1:1 allocation ratio pre-operatively, stratified by centre.

#### Allocation concealment mechanism

The randomisation sequence will be concealed using several measures including using an automated randomisation system and restricting access to the randomisation sequence. The randomisation sequence will be saved in a restricted section of the trial master file which will only be accessible by statisticians and not those who enrol or assign interventions.

### Study drug

#### Study drug description

Simvastatin is a HMG-CoA reductase inhibitor which also has anti-inflammatory activity. The comparator is a matched placebo tablet with no active ingredient. Placebo is used as a comparator because of the lack of any preventative pharmacotherapy demonstrated to be beneficial for this condition. The study drug will be in the form of tablet which will be taken orally or through a feeding tube (if placed) for 4 days before surgery and for up to 7 days post surgery. The duration of the therapy is based on the anti-inflammatory effects of simvastatin 80 mg in the human inhaled LPS model of pulmonary injury [12] and the reduction in pulmonary inflammation in the single-centre proof-of-concept study in oesophagectomy patients [14] utilising this dose and duration. The study drug will be dispersed in 10–20 mL of water when administered via the feeding tube.

#### Study drug supply

Patient drug packs will be prepared by Victoria Pharmaceuticals. Simvastatin 40 mg or matching placebo tablets will be packaged in a white, opaque, high-density polyethylene plastic container which will be sealed with a tamper-evident seal and labelled in compliance with applicable regulatory requirements. Each container will contain 40 tablets of study drug for the treatment of one patient for 20 days (which includes 9 days’ coverage to allow for surgical delays and drug wastage by accident). All trial drugs will be packaged identically and identified only by a unique medication pack identification number in accordance with the study randomisation sequence.

#### Study drug termination

The study drug will be discontinued if any one of the following conditions is met, prior to the maximum treatment period (11 days from the start of study drug):

1. Study-drug-related AE:
  - CK > 10 times the ULN of the local laboratory range
  - ALT/AST > 5 times the ULN of the local laboratory range
2. Development of a clinical condition requiring immediate treatment with a statin or other drugs which interact with statins
3. Discontinuation of active medical treatment
4. Patient’s request for withdrawal from the study
5. Decision by the attending clinician that the study drug should be discontinued on safety grounds
6. Discharge from hospital
7. Change of type of surgery
8. Death

### Withdrawal of consent post randomisation

Patients may withdraw or be withdrawn from the trial at any time without prejudice. Data recorded up to the point of withdrawal will be included in the trial analysis, unless consent to use their data has also been withdrawn. If a patient requests termination of the trial drug during the treatment period, the drug will be stopped but the patient will continue to be followed-up as part of the trial. If a patient withdraws consent during trial treatment, the trial drug will be stopped but permission will be sought to access medical records for data related to the trial. If a patient wishes to withdraw from the trial after completion of trial treatment, permission to access medical records for trial data will be sought.

### Intervention adherence

Adherence to the study drug will be monitored by recording the number of tablets returned at the end of the treatment period which will be used to calculate the number of doses administered. The patients will also maintain a diary record of self-administration prior to admission for their surgery. Patients will be withdrawn from the study if they have taken no study drug at all prior to surgery.

### Blinding

This is a double-blind, placebo-controlled trial, and patients, clinicians and the study team will be blinded to each patient’s treatment allocation. All trial drugs, whether simvastatin or placebo, will be packaged identically and identified only by a unique, medication-pack identifier.

### Emergency unblinding

Emergency unblinding can be requested by a PI or designated investigator on safety grounds, or if the treatment decision for a patient could be influenced by the knowledge of what the patient is taking as part of the trial. If the PI or designated investigator decides that there is justification to unblind a patient, emergency unblinding can be performed via the randomisation system. In the event of failure of the online system, back-up manual unblinding will be performed by the clinical trials pharmacist between 9 a.m. and 5 p.m. or the on-call pharmacist out of hours in the Royal Victoria Hospital, Belfast. In the event that unblinding occurs, the patient may discontinue the study drug but will remain in the trial unless they decide to withdraw. Where unblinding has occurred, this should be fully documented by the site and the Clinical Trials Unit (CTU) informed.

### Data collection

To ensure that accurate, complete and reliable data are collected, the CTU will provide training to site staff in the format of investigator meetings and/or site-initiation visits. Data will be collected through electronic data capture (EDC). Data will be obtained through patient’s hospital notes, hospital laboratory records and image records. All data for an individual patient will be collected by the PI or their delegated nominees and recorded in the EDC database for the study except 90-day follow-up data. For the economic evaluation, HRQoL will be measured by the EQ-5D-5 L at baseline and at 90 days post surgery. Resource use data will be collected via the questionnaire administered at 90 days post surgery. The patients’ confidentiality will be maintained and will not be made publicly available to the extent permitted by the applicable laws and regulations. On completion of the trial, the trial master file will be archived by the CTU and the investigator site file and study data will be archived by the PI at each site according to the applicable regulatory requirements.

### Follow-up visits and procedures

All survivors will be followed up at 90 days after surgery. HRQoL will be measured using the EQ-5D-5 L administered at baseline and at 90 days post surgery. Health and social care resource use will be collected via a questionnaire at 90 days post surgery. Where the patient has been discharged from hospital, questionnaires will be administered via post. If questionnaires are not returned, a maximum of two telephone contacts will be made to the study participant; the first call will check that the questionnaire has been received and the participant is happy to complete it. If necessary, a second copy of the questionnaire will be sent. In the event of non-return, one further telephone contact will be made and the health economic data collected over the telephone where possible. To minimise the risk of causing distress by contacting relatives of patients who have since deceased, the CTU will contact the patient’s GP to ascertain the patient survival status prior to any contact being made.

### Safety monitoring and adverse event (AE) reporting

The AEs will be defined as stipulated by the Medicine and Healthcare Products Regulatory Agency (MHRA) Good Clinical Practice Guide 2012 and reported as per guidance. The PI or designee will record all directly observed AEs and those reported by the patient. In addition, the patient will be asked about AEs up to 28 days post surgery or on discharge from hospital if earlier. The PI or designee must assess all AEs for seriousness, causality, severity and if the AE is related to the study drug for expectedness. All reportable AEs should be recorded in the patient’s medical notes and on the AE form within the EDC.

Prevention HARP-2 is recruiting a population that is already undergoing a major surgical intervention, it is expected that many of the participants will experience AEs. Events that are expected in this population (i.e. events that are in keeping with the patient’s underlying medical or surgical condition) should not be reported as AEs or SAEs. Death which is ascertained to be not related to surgical complications or progression of the primary or secondary outcomes should be reported as a SAE. SAEs will be evaluated by the PI for causality (i.e. their relationship to study drug) and expectedness. An adverse reaction (AR) is an AE which is related to the administration of the study drug. If any AEs are related to the study drug (i.e. are ARs) they must be reported on the AE form within the EDC.

The following are ARs which are expected and must be reported on the AE form within the EDC: CK > 10 times the ULN, ALT/AST > 5 times the ULN.

The following serious adverse reaction is expected and must be reported on the SAE form within the Case Report Form (CRF): need for renal replacement therapy in patients with CK > 10 times the ULN.

All SAEs should be reported within 24 h of becoming aware of their occurrence. They should be reported to the CTU and the chief investigator (CI), who will inform the sponsor and regulatory authorities.

### Statistical analysis plan

This is a randomised, placebo-controlled, parallel-group, superiority trial. Intention-to-treat analysis (ITT) will be followed for the primary analysis and patients will be analysed according to the treatment that they were randomised to, disregarding the actual treatment received. Patients should have received at least one dose pre surgery for inclusion into the analysis. Patients who did not proceed to the planned surgery will also be excluded from the final analysis. A per-protocol analysis will be performed in addition to the ITT analysis.

The primary outcome measure will be compared between treatment groups using a chi-square test. We will also report each primary outcome component separately. A secondary analysis will involve a logistic regression model, with the dependent variable as composite endpoint/no composite endpoint within 7 days and the independent variables as treatment, centre, type of surgery and age as a covariate. An odds ratio measuring the treatment effect and its 95% confidence interval will be reported. Other categorical outcomes will be analysed using logistic regression models, with treatment group as an independent variable along with centre, type of surgery and age as a covariate. The summary statistics will be based on proportions and the 95% confidence interval. Continuous outcomes will be analysed using linear regression models, with treatment group as an independent variable and terms for centre, type of surgery and age in the model. Difference in treatment will be based on adjusted mean estimates and 95% confidence intervals. Time-to-event data will be analysed using a log-rank test. Any patients who have not experienced an event at the time point of interest or withdrawn will be censored. The proportion experiencing an event over time will be illustrated using a Kaplan-Meier curve for each of the treatment groups. The *p* values and a hazard ratio with its 95% confidence interval from a Cox proportional hazards model will also be presented. The proportional hazard assumption across treatment arms will be checked graphically using a log-cumulative hazard plot.

Baseline characteristics, follow-up measurements and safety data will be described using appropriate descriptive summary measures depending on the scale of measurement and distribution.

Subgroup analyses will use a statistical test for interaction and will be reported using 99% confidence interval. Four subgroup analyses are pre-specified, stratifying by chemotherapy prior to surgery (yes/no), type of surgery (oesophagectomy, lobectomy, pneumonectomy), surgical technique (minimally invasive/hybrid/open) and duration of OLV (≤ 120 min and > 120 min [20]). We shall also investigate subgroups based on smoking status.

An interim analysis will also be conducted to analyse efficacy and safety parameters. This will occur when approximately 50% of the planned number of patients to be randomised have completed day 28 assessment or have been discharged from hospital, whichever is sooner. In relation to efficacy, a chi-square test will be applied with a *p* value < 0.001 according to the Haybittle-Peto stopping rule. For safety, the acute kidney injury data will be presented alongside other safety data.

Every effort will be made to minimise missing baseline and outcome data in this trial. The level and pattern of the missing data in the baseline variables and outcomes will be established by forming appropriate tables and the likely causes of any missing data will be investigated. This information will be used to determine whether the level and type of missing data have the potential to introduce bias into the analysis results for the proposed statistical methods, or substantially reduce the precision of estimates related to treatment effects. If necessary, these issues will be dealt with using multiple imputation or Bayesian methods for missing data as appropriate. A detailed statistical analysis plan will be produced prior to the end of patient recruitment and database lock.

### Health economic evaluation

A within-trial cost-utility analysis will be conducted to assess the cost-effectiveness of simvastatin compared to placebo at 90 days’ follow-up (post-surgery). A health service perspective will be adopted as recommended by the National Institute for Health and Care Excellence (NICE) [37]. The health outcome for the analysis will be quality-adjusted life years (QALYs) and these will be calculated for each patient using responses on the EQ-5D-5 L. Patient-level healthcare resource use (of primary, community and social care services) will be obtained from the trial CRF and the self-completed patient questionnaires, and combined with publicly available unit costs to estimate costs for each participant. It will not be necessary to discount costs and outcomes given the duration of follow-up. Standard methods will be used to explore and display uncertainty in the cost-effectiveness data including scatter plots on the cost-effectiveness plane and cost-effectiveness acceptability curves. Sensitivity analyses will be performed to assess the robustness of the cost-effectiveness results to changes in key parameters. A detailed health economic analysis plan will be finalised prior to commencing the analysis.

### Trial organisation/oversight

Participant safety will be monitored by an independent Data Monitoring and Ethics Committee through regular review of AEs. A Trial Steering Committee will provide trial oversight. The members of the Committees will be experienced in clinical trials and will be from a variety of backgrounds including clinical trialists, a statistician and a lay public member. All protocol amendments will be implemented only after approval from the sponsor, Ethics Committee and MHRA. The trial will comply with the principles of GCP, the requirements and standards set out by the EU Directive 2001/20/EC and the applicable regulatory requirements in the UK, the Medicines for Human Use (Clinical Trials) Regulations 2004 and subsequent amendments and the Research Governance Framework. It will also adhere to the SOPs of the NICTU. The trial will be subject to independent audit initiated by the sponsor, MHRA and trial monitoring as per NICTU monitoring SOPs.

### Dissemination

The results of the trial will initially be reported to the trial collaborators and the findings will be presented at national and international meetings with open-access abstracts on-line, e.g. the American Thoracic Society annual meeting. The findings will be published in high-quality, peer-reviewed, open-access (via PubMed) journals in accordance with the open-access policies and make the results readily accessible to the public, healthcare professionals and scientists. A lay person’s summary of the principal findings of the results will be sent to all patients involved in the study at their request. In addition, a lay person’s summary will be sent to local and national patient support and liaison groups (e.g. ICU Steps, hospital patient groups). A report of the study findings will be sent to the INVOLVE registry. Where appropriate, research details will also be posted on institutional websites available to the general public. In addition, the most significant results will be communicated to the public through press releases. The sponsor and funder do not have any involvement in the trial methodology, data collection, analysis and dissemination.

### Trial status and summary

Prevention HARP 2 is a UK multi-centre, randomised trial comparing peri-operative simvastatin 80 mg and placebo in preventing PPC and MI in adult patients undergoing OLV for oesophagectomy, lobectomy and pneumonectomy. The current protocol version is v7.0 dated 7 December 2017. The recruitment to this trial commenced in November 2016 and is expected to complete in July 2020.

## Additional file

Additional file 1: Standard Protocol Items: Recommendations for Interventional Trials (SPIRIT) 2013 Checklist: recommended items to address in a clinical trial protocol and related documents*. (PDF 86 kb)

## Abbreviations

AE: Adverse event
AF: Atrial fibrillation
ALT: Alanine aminotransferase
AR: Adverse reaction
ARDS: Acute respiratory distress syndrome
AST: Aspartate aminotransferase
BHSCT: Belfast Health and Social Care Trust
CI: Chief investigator
CK: Creatine kinase
CONSORT: Consolidated Standards of Reporting Trials
CrCl: Creatinine clearance
CRF: Case Report Form
CTU: Clinical Trials Unit
EDC: Electronic data capture
EQ-5D-5 L: EuroQoL-5 Dimension Questionnaire (5-level version)
GCP: Good Clinical Practice
HRQoL: Health-related quality of life
ICU: Intensive care unit
ISRCTN: International Standard Randomised Controlled Trial Number Register
ITT: Intention-to-treat analysis
MGS: Melbourne Group Scale
MI: Myocardial infarction
MINS: Myocardial ischaemia post non-cardiac surgery
NICE: National Institute for Health and Care Excellence
NICTU: Northern Ireland Clinical Trials Unit
NIHR: National Institute for Health Research
OLV: One-lung ventilation
PI: Principal investigator
PPC: Post-operative pulmonary complications
QALY: Quality-adjusted life year
SAE: Serious adverse event
SOPs: Standard operating procedures
SrCr: Serum creatinine
SUSAR: Suspected unexpected serious adverse reaction
ULN: Upper limit of normal
